# Supplementary material for: Modeling glioblastoma heterogeneity as a dynamic network of cell states
Source: Mol Syst Biol. 2021 Sep 16;17(9):e10105. doi: 10.15252/msb.202010105 (PMC8444284; doi:10.15252/msb.202010105)
Supplement: Supplementary file 6 — Source Data for Figure 5 [file MSB-17-e10105-s004.zip › Figure5A_sourcedata/GSEA_3071/nosigdb_stateIII.GseaPreranked.1622795155562/LARSSON_STATE_3.html]

Details for gene set LARSSON\_STATE\_3[GSEA]

|  || Dataset | state33071 |
| Phenotype | NoPhenotypeAvailable |
| Upregulated in class | na\_neg |
| GeneSet | LARSSON\_STATE\_3 |
| Enrichment Score (ES) | -0.621008 |
| Normalized Enrichment Score (NES) | -3.3292682 |
| Nominal p-value | 0.0 |
| FDR q-value | 0.0 |
| FWER p-Value | 0.0 |
Table: GSEA Results Summary

  

Fig 1: Enrichment plot: LARSSON\_STATE\_3      
 Profile of the Running ES Score & Positions of GeneSet Members on the Rank Ordered List

  

| PROBE | GENE SYMBOL | GENE\_TITLE | RANK IN GENE LIST | RANK METRIC SCORE | RUNNING ES | CORE ENRICHMENT || 1 | SLC44A1 |  |  | 1119 | -0.286 | -0.2783 | No |
| 2 | LAMC1 |  |  | 1120 | -0.286 | -0.2683 | No |
| 3 | TFPI2 |  |  | 1128 | -0.286 | -0.2601 | No |
| 4 | GGH |  |  | 1439 | -0.302 | -0.3294 | No |
| 5 | CALR |  |  | 1498 | -0.306 | -0.3337 | No |
| 6 | WLS |  |  | 1517 | -0.307 | -0.3276 | No |
| 7 | TIMP1 |  |  | 2038 | -0.337 | -0.4498 | No |
| 8 | DSEL |  |  | 2180 | -0.347 | -0.4739 | No |
| 9 | CANX |  |  | 2209 | -0.349 | -0.4689 | No |
| 10 | BSG |  |  | 2316 | -0.359 | -0.4837 | No |
| 11 | IQGAP1 |  |  | 2405 | -0.366 | -0.4936 | No |
| 12 | PDIA6 |  |  | 2505 | -0.373 | -0.5060 | No |
| 13 | DYNC1H1 |  |  | 2580 | -0.380 | -0.5118 | No |
| 14 | ATP6AP2 |  |  | 2591 | -0.381 | -0.5010 | No |
| 15 | SPTBN1 |  |  | 2647 | -0.385 | -0.5017 | No |
| 16 | HMGCR |  |  | 2673 | -0.388 | -0.4946 | No |
| 17 | LMAN1 |  |  | 2743 | -0.394 | -0.4986 | No |
| 18 | ALCAM |  |  | 2853 | -0.403 | -0.5125 | No |
| 19 | GPM6A |  |  | 3171 | -0.440 | -0.5788 | No |
| 20 | HSPA5 |  |  | 3193 | -0.442 | -0.5688 | No |
| 21 | CD63 |  |  | 3258 | -0.452 | -0.5694 | No |
| 22 | ITM2B |  |  | 3314 | -0.463 | -0.5674 | No |
| 23 | SYNE2 |  |  | 3523 | -0.518 | -0.6029 | Yes |
| 24 | CALU |  |  | 3566 | -0.533 | -0.5951 | Yes |
| 25 | RPN2 |  |  | 3581 | -0.539 | -0.5798 | Yes |
| 26 | DST |  |  | 3597 | -0.545 | -0.5646 | Yes |
| 27 | PLAT |  |  | 3600 | -0.547 | -0.5460 | Yes |
| 28 | SQLE |  |  | 3606 | -0.553 | -0.5279 | Yes |
| 29 | CD9 |  |  | 3634 | -0.567 | -0.5150 | Yes |
| 30 | FGFBP3 |  |  | 3649 | -0.578 | -0.4984 | Yes |
| 31 | TOP2A |  |  | 3671 | -0.594 | -0.4831 | Yes |
| 32 | SOX6 |  |  | 3675 | -0.596 | -0.4630 | Yes |
| 33 | FAT1 |  |  | 3685 | -0.604 | -0.4442 | Yes |
| 34 | APLP2 |  |  | 3693 | -0.610 | -0.4246 | Yes |
| 35 | GRIA2 |  |  | 3721 | -0.633 | -0.4095 | Yes |
| 36 | MMP16 |  |  | 3753 | -0.675 | -0.3938 | Yes |
| 37 | HSP90B1 |  |  | 3757 | -0.681 | -0.3708 | Yes |
| 38 | APP |  |  | 3779 | -0.710 | -0.3513 | Yes |
| 39 | MKI67 |  |  | 3788 | -0.721 | -0.3282 | Yes |
| 40 | NCAM1 |  |  | 3821 | -0.773 | -0.3093 | Yes |
| 41 | CENPF |  |  | 3842 | -0.820 | -0.2858 | Yes |
| 42 | PIF1 |  |  | 3844 | -0.824 | -0.2572 | Yes |
| 43 | KCNQ1OT1 |  |  | 3866 | -0.885 | -0.2317 | Yes |
| 44 | SCRG1 |  |  | 3869 | -0.904 | -0.2006 | Yes |
| 45 | PTN |  |  | 3884 | -1.022 | -0.1684 | Yes |
| 46 | POSTN |  |  | 3885 | -1.025 | -0.1326 | Yes |
| 47 | ASPM |  |  | 3899 | -1.166 | -0.0951 | Yes |
| 48 | PTPRZ1 |  |  | 3913 | -1.342 | -0.0515 | Yes |
| 49 | MALAT1 |  |  | 3924 | -1.583 | 0.0013 | Yes |
Table: GSEA details [plain text format]

  

Fig 2: LARSSON\_STATE\_3: Random ES distribution      
 Gene set null distribution of ES for **LARSSON\_STATE\_3**
